# Supplementary material for: Allogenic Adipose-Derived Stem Cells in Diabetic Foot Ulcer Treatment: Clinical Effectiveness, Safety, Survival in the Wound Site, and Proteomic Impact
Source: Int J Mol Sci. 2023 Jan 12;24(2):1472. doi: 10.3390/ijms24021472 (PMC9864558; doi:10.3390/ijms24021472)
Supplement: Supplementary file 1 [file ijms-24-01472-s001.zip › Online-Only Supplementary Material after revision.docx]

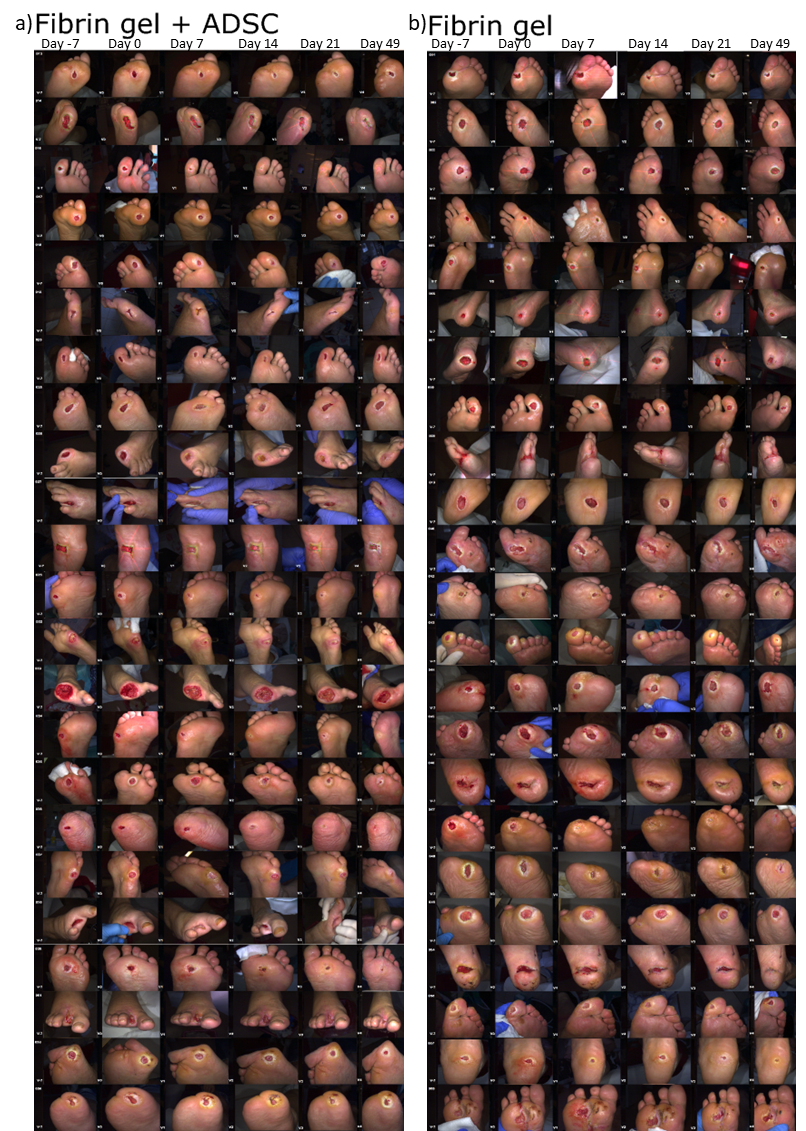


Figure S1 Photographic documentation of the wound from the complete set of patients that underwent the whole course of this clinical study. The photographs were taken during the visits on days -7, 0, 7, 14, 21 and 49.

Panel a) – photographs of the wounds of patients from ADSC group. Panel b) – photographs of the wounds of patients from fibrin gel group.


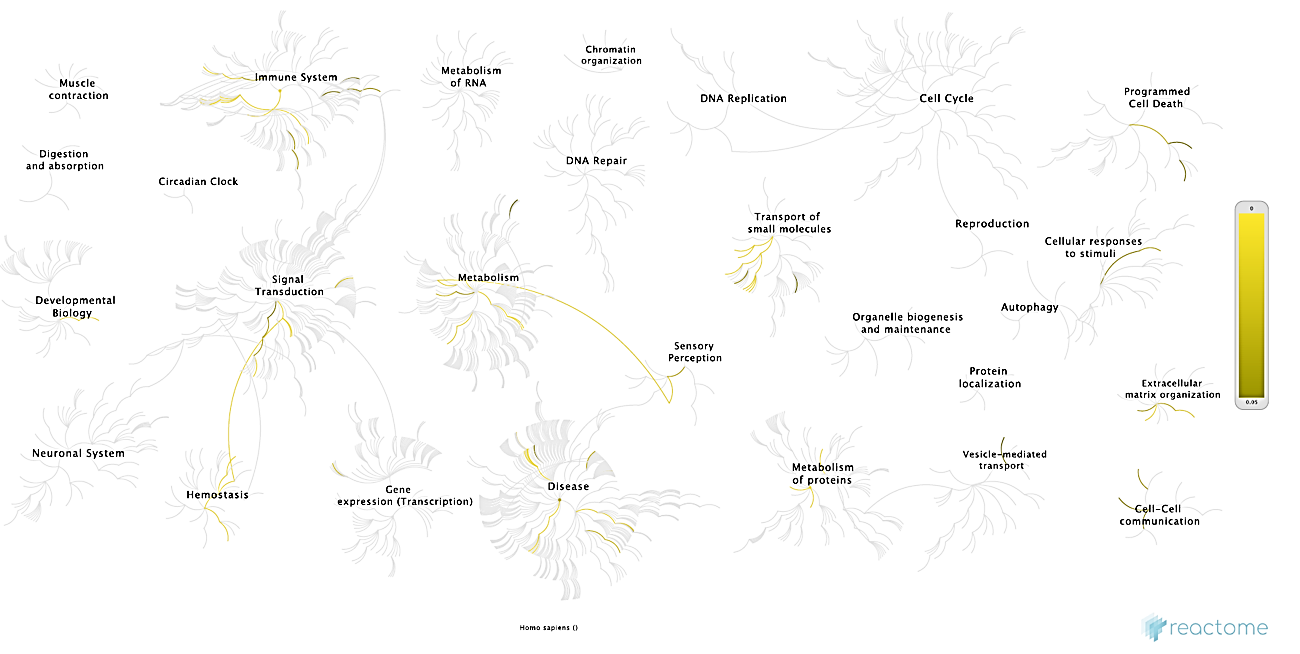


**Figure S2** Possible metabolic pathways activated upon ADSC admission to the wound. Metabolic pathways predicted to be active in wound scrapings based on bioinformatic analysis of overexpressed proteins in wound scrapings from ADSC group patients compared to fibrin gel group patients. Analysis was performed in reactome.org online database


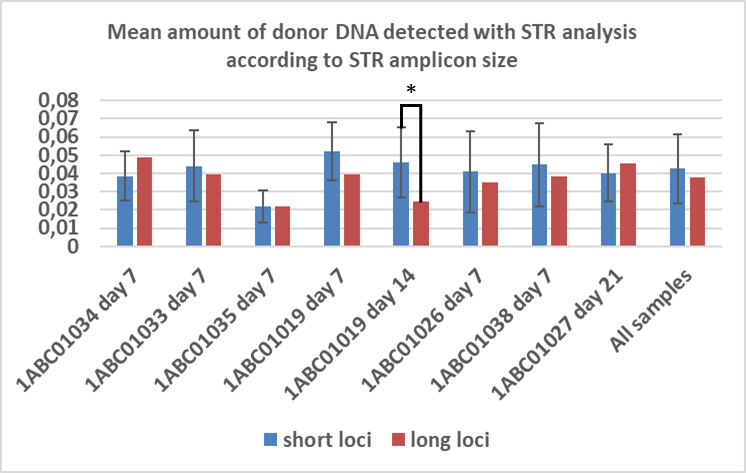


**Figure S3** Comparison of STR amplicon size. Mean amount of donor DNA detected with STR (short tandem repeats) analysis according to STR amplicon size *p=0,0041, student t-test, vertical bars indicate standard deviation (SD).


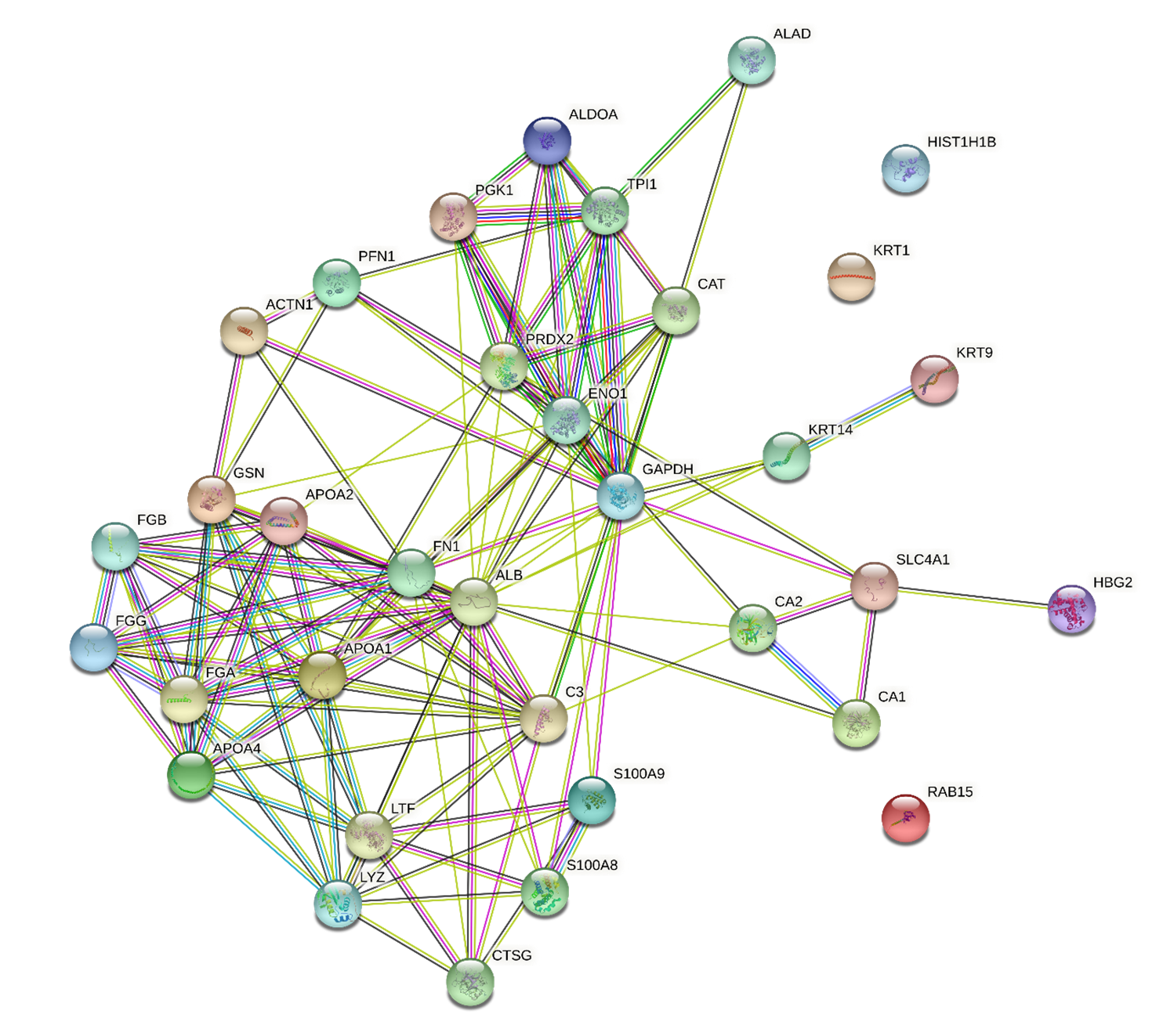


**Figure S4** String pathway analysis based on proteomic results. To verify if the proteins identified as upregulated on day 7 in the ADSC group vs day 0 in ADSC and day 7 in the control group, are functionally related or if they are random changes, we analyzed the network of interactions between the upregulated proteins using the STRING-db software. FDR (false discovery rate) calculated for that network of interaction is 1.0e-16, which indicates their close functional relationship, and thus the proteins are not random identifications.

**Table S1** Evaluation of pain sensation assessed by Visual Analog Scale in ADSC + Fibrin gel and Fibrin gel groups. Pain was assessed on a scale from 0 to 100. The Wilcoxon test was used to compare pain intensity in the groups.

| Day | ADSC + Fibrin gel  (n = 23) median  min, max | Fibrin gel  (n= 23) median  min, max | p value |
| --- | --- | --- | --- |
| -7 | 10.0  0.0, 50.0 | 5.0  0.0, 50.0 | 0.420 |
| 0 | 10.0  0.0, 55.0 | 10.0  0.0, 70.0 | 0.648 |
| 7 | 10.0  0.0, 50.0 | 0.0  0.0, 80.0 | 0.542 |
| 14 | 0.0  0.0, 40.0 | 0.0  0.0, 40.0 | 0.728 |
| 21 | 0.0  0.0, 60.0 | 0.0  0.0, 60.0 | 1.000 |
| 49 | 0.0  0.0, 45.0 | 0.0  0.0, 50.0 | 0.617 |

**Table S2** Evaluation of adverse events in ADSC + Fibrin gel and Fibrin gel groups. To verify whether the number of adverse events was different in two groups two sample poisson test was used

| **Adverse event** | Fibrin Gel + ADSC | Fibrin Gel | p value |
| --- | --- | --- | --- |
| Blood pressure increased | 4 | 6 | 0.75 |
| Edema of lower limb | 0 | 3 | 0.25 |
| Neuropathic pain | 1 | 3 | 0.63 |
| Cellulitis | 1 | 0 | 1.0 |
| TOTAL | 6 | 12 | 0.24 |

.

**Table S3** List of 34 proteins identified in wound scrapings of ADSC group with elevated expression levels in day 7 compared to day 0. Detailed information on function, role in wound healing and confirmed or putative AMP activity of 34 proteins identified in wound scrapings from ADSC group patients with elevated expression levels in day 7 compared to day 0.

| **No.** | **Protein name** | **Gene name** | **Uniprot access number** | **Function** | **Literature-based connection to wound healing processes** | **Wound healing literature** | **AMP activity or probability** | **AMP literature** |
| --- | --- | --- | --- | --- | --- | --- | --- | --- |
| 1 | Protein S100-A8 | S100A8 | P05109 | Calprotectin | Stimulation of MSC to improve wound healing | [1] | Confirmed AMP activity | [2] |
|  |  |  |  | Calcium-binding |  |  |  |  |
|  |  |  |  | Calcium homeostasis regulation, |  |  |  |  |
| 2 | Protein S100-A9 | S100A9 | P06702 | Partake in signaling pathways involved in inflammatory process | Antimicrobial proteins | [3] |  |  |
|  |  |  |  | Expressed by myeloid cells; |  | [4] |  |  |
|  |  |  |  | Zinc and manganese chelator, which inhibits microbial growth. |  |  |  |  |
| 3 | Glyceraldehyde-3-phosphate dehydrogenase | GAPDH | P04406 | Enzyme in the glycolysis pathway. | Polarisation of macrophages from M1 to M2 phenotype (extracellular function). | [5] | 0.7626 probability of AMP activity |  |
|  |  |  |  |  |  |  |  |  |
| 4 | Alpha-Actinin -1 | ACTN1 | P12814 | Determines the movement of keratinocytes. | Necessary for epidermination . | [6] | Not reported |  |
| 5 | Peroxiredoxin II | PRDX2 | P32119 | ROS elimination. | It protected MSC from ROS-induced apoptosis, what enhanced wound healing . | [7] | Not reported |  |
| 6 | Lactotransferrin | LTF | E7ER44 | Fe-binding protein. | Stimulates proliferation and migration of fibroblasts and keratinocytes and increases synthesis of ECM components. | [8] | Confirmed AMP activity | [9] |
|  |  |  |  |  | Antimicrobial protein |  |  |  |
| 7 | Gelsolin (Actin-depolymerizing factor) | GSN | A0A0A0MS51 | Actin filament binding. | Analgesic and anti-inflammatory effects (mouse model). | [10] | Not reported |  |
|  |  |  |  |  | Fibroblasts lacking gelsolin showed a considerable defect in the healing process | [11] |  |  |
| 8 | Lysozyme C | LYZ | P61626 | Bacteriolytic function. | Antimicrobial protein | [12] | Confirmed AMP activity | [13] |
|  |  |  |  |  | Markedly increases re-epithelialization, fibrogenesis and neovascularization |  |  |  |
| 9 | Fibrinogen alpha chain | FGA | P02671 | Main proteins of the thrombus, preventing blood loss | Fibrin matrix facilitates cell adhesion, migration and proliferation, and thus stimulates angiogenesis | [14], [15] | Not reported |  |
|  |  |  |  |  |  |  |  |  |
| 10 | Fibrinogen beta chain | FGB | P02675 |  |  |  |  |  |
|  |  |  |  |  |  |  |  |  |
| 11 | Fibrinogen gamma chain | FGG | C9JEU5 |  |  |  |  |  |
|  |  |  |  |  |  |  |  |  |
| 12 | Histone H1.5 | H1-5 | P16401 | Binds to linker DNA between nucleosomes. | Possible antimicrobial function. | [16] | 0.9126 probability of AMP activity |  |
|  |  |  |  |  |  |  |  |  |
|  |  |  |  |  |  |  |  |  |
| 13 | Albumin | ALB | P02768 | Blood transport protein. | Patients with lower serum albumin concentration had greater risk of amputation in diabetic foot ulcer. | [17] | Not reported |  |
|  |  |  |  |  |  |  |  |  |
|  |  |  |  |  |  |  |  |  |
|  |  |  |  |  |  |  |  |  |
|  |  |  |  |  |  |  |  |  |
| 14 | Profilin-1 | PFN1 | P07737 | Binds actin and has an impact on cytoskeleton structure. | Proangiogenic function – regulates migration, invasion and capillary morphogenesis (studied on HUVEC) | [18] | Not reported |  |
|  |  |  |  |  |  |  |  |  |
|  |  |  |  |  |  |  |  |  |
|  |  |  |  |  |  |  |  |  |
|  |  |  |  |  |  |  |  |  |
| 15 | Carbonic anhydrase 1 | CA1 | P00915 | Reversible hydration of carbon dioxide to carbonic acid. | Expressed in epidermis and papillary layer of the skin. It plays an important role in wound healing and re-epithelialisation | [19] | 0.72 probability of AMP activity |  |
| 16 | Carbonic anhydrase 2 | CA2 | P00918 |  | Can be found in keratinocytes. | [20] | Not reported |  |
|  |  |  |  |  | CA2 levels in bioptates can be related to increased keratinocytes presence in wound site |  |  |  |
| 17 | Catalase | CAT | P04040 | ROS elimination. | Essential for the transition from inflammation to proliferation phase during wound healing. | [21] | Not reported |  |
|  |  |  |  |  |  |  |  |  |
|  |  |  |  |  |  |  |  |  |
| 18 | Keratin type II cytoskeletal 1 | KRT1 | P04264 | It can be found in differentiated keratinocytes | Keratin dressings accelerate wound healing and wound closure | [22] | Not reported |  |
|  |  |  |  |  |  |  |  |  |
|  |  |  |  |  |  |  |  |  |
| 19 | Keratin type I cytoskeletal 9 | KRT9 | P35527 | Present in palmoplantar epidermis. |  | [23] | Not reported |  |
|  |  |  |  |  |  |  |  |  |
| 20 | Keratin. type I cytoskeletal 14 | KRT14 | P02533 | Expressed in basal layers of keratinocytes. |  | [24] | Not reported |  |
|  |  |  |  |  |  |  |  |  |
| 21 | Band 3 anion transport protein | SLC4A1 | P02730 | Membrane transport of inorganic anions. | It can be found in human skin. | [25] | Not reported |  |
|  |  |  |  |  |  |  |  |  |
| 22 | Triosephosphate isomerase | TPI1 | P60174 | Enzyme in the glycolysis pathway. | Studies showed increased gene expression in cells involved in wound healing and hMSC cultured in hypoxic conditions. | [26] | 0.7491 probability of AMP activity |  |
|  |  |  |  |  |  |  |  |  |
|  |  |  |  |  |  |  |  |  |
|  |  |  |  |  |  |  |  |  |
| 23 | Cathepsin G | CTSG | P08311 | Serine protease with specificity similar to trypsin and chymotrypsin. Cleaves complement C3. Exhibits antibacterial activity against the Gram-negative bacterium P.aeruginosa. | High activity in chronic wounds may inhibit healing processes through degradation of growth factors, cytokines ECM proteins | [27] | Confirmed AMP activity | [28] |
|  |  |  |  |  |  |  |  |  |
|  |  |  |  |  |  |  |  |  |
|  |  |  |  |  |  |  |  |  |
|  |  |  |  |  | Cathepsin G is involved in platelet activation, internalized pathogens clearance and chemokine modification processes |  |  |  |
|  |  |  |  |  |  |  |  |  |
|  |  |  |  |  |  |  |  |  |
|  |  |  |  |  |  |  |  |  |
|  |  |  |  |  |  |  |  |  |
| 24 | Complement C3 | C3 | P01024 | C3 plays a major role in the activation of the complement system. | Contradictory literature data: | [29] | Not reported |  |
|  |  |  |  |  | Beneficial effects observed in C3-treated mice: increased influx of inflammatory cells, fibroblast migration and collagen deposition |  |  |  |
|  |  |  |  |  |  |  |  |  |
|  |  |  |  |  |  |  |  |  |
|  |  |  |  |  |  |  |  |  |
|  |  |  |  |  |  |  |  |  |
|  |  |  |  |  | Impairing effect: C3 knock-out mice healed faster than wild type mice during early wound healing phases. | [30] |  |  |
|  |  |  |  |  |  |  |  |  |
|  |  |  |  |  |  |  |  |  |
|  |  |  |  |  |  |  |  |  |
| 25 | Alpha-enolase | ENO1 | P06733 | Enzyme in the glycolysis pathway | Glycolysis is the main energy source for inflammatory macrophages. | [31] | Not reported |  |
| 26 | Phosphoglycerate kinase 1 | PGK1 | P00558 |  |  |  | 0.7131 probability of AMP activity |  |
|  |  |  |  |  | Repairing epithelium derives in 70% Energy from glycolysis, and only 30% from Krebs cycle | [32] |  |  |
|  |  |  |  |  |  |  |  |  |
| 27 | Fructose-bisphosphate aldolase | ALDOA | H3BQN4 |  |  |  | 0.7489 probability of AMP activity |  |
|  |  |  |  |  |  |  |  |  |
|  |  |  |  |  |  |  |  |  |
| 28 | Fibronectin | FN1 | P02751 | Main ECM component. | It mediates keratinocyte migration across the wound bed. | [33] | Not reported |  |
|  |  |  |  |  |  |  |  |  |
|  |  |  |  |  | It provides matrix for collagen deposition during early wound healing phases. | [34] |  |  |
|  |  |  |  |  |  |  |  |  |
|  |  |  |  |  |  |  |  |  |
|  |  |  |  |  | When degradation of fibronectin matrix is disturbed, healing is impaired and the acute wound changes to a chronic wound. | [35] |  |  |
|  |  |  |  |  |  |  |  |  |
|  |  |  |  |  |  |  |  |  |
|  |  |  |  |  |  |  |  |  |
| 29 | Apolipoprotein A-I | APOA1 | P02647 | HDL component. | Anti-inflammatory and anti-atherosclerotic effects. | [36] | Not reported |  |
|  |  |  |  |  |  |  |  |  |
| 30 | Apolipoprotein A-IV | APOA4 | P06727 | HDL and chylomicron component. | Anti-inflammatory effect (experimental colitis model). | [37] | Not reported |  |
|  |  |  |  |  |  | [38] |  |  |
| 31 | Hemoglobin subunit gamma 2 | HBG2 | P69892 | Oxygen transport. | Pressure ulcers of patients with higher hemoglobin tend to heal better than with lower hemoglobin. | [39] | 0.7346 probability of AMP activity |  |
|  |  |  |  |  |  |  |  |  |
|  |  |  |  |  |  |  |  |  |
|  |  |  |  |  |  |  |  |  |
|  |  |  |  |  | It may mean better tissue oxygenation |  |  |  |
|  |  |  |  |  |  |  |  |  |
| 32 | Delta-aminolevulinic acid dehydratase | ALAD | P13716 | Catalyzes synthesis of porphobilinogen. | No literature evidence of a connection to wound healing. |  | Not reported |  |
|  |  |  |  |  |  |  |  |  |
| 33 | Apolipoprotein A II | APOA2 | P02652 | Second most abundant protein building HDL. |  |  | Not reported |  |
|  |  |  |  |  |  |  |  |  |
| 34 | Ras-related protein Rab-15 | RAB15 | A0A2R8YDI9 | GTPase activity. |  |  | Not reported |  |
|  |  |  |  |  |  |  |  |  |
| 35 | Histone H4 | H4C1 | P62805 | Core component of nucleosome |  |  | 0.9341 probability of AMP activity |  |
|  |  |  |  |  |  |  |  |  |
|  |  |  |  |  |  |  |  |  |
| 36 | Histone H3.1 | H3C1 | P68431 |  |  |  | 0.7185 probability of AMP activity |  |
|  |  |  |  |  |  |  |  |  |
|  |  |  |  |  |  |  |  |  |

**References:**

1. Basu, Abhijit, Saira Munir, Medanie A Mulaw, Karmveer Singh, Diana Crisan, Anca Sindrilaru, Nicolai Treiber, Meinhard Wlaschek, Markus Huber-Lang, and Florian Gebhard. "A Novel S100a8/A9 Induced Fingerprint of Mesenchymal Stem Cells Associated with Enhanced Wound Healing." *Scientific reports* 8, no. 1 (2018): 1-16.

2. Sroussi, Herve Y, Gerwald A Köhler, Nina Agabian, Dana Villines, and Joel M Palefsky. "Substitution of Methionine 63 or 83 in S100a9 and Cysteine 42 in S100a8 Abrogate the Antifungal Activities of S100a8/A9: Potential Role for Oxidative Regulation." *FEMS Immunology & Medical Microbiology* 55, no. 1 (2009): 55-61.

3. Kehl-Fie, Thomas E, Seth Chitayat, M Indriati Hood, Steven Damo, Nicole Restrepo, Carlos Garcia, Kim A Munro, Walter J Chazin, and Eric P Skaar. "Nutrient Metal Sequestration by Calprotectin Inhibits Bacterial Superoxide Defense, Enhancing Neutrophil Killing of Staphylococcus Aureus." *Cell host & microbe* 10, no. 2 (2011): 158-64.

4. Shabani, Fatemeh, Alireza Farasat, Majid Mahdavi, and Nematollah Gheibi. "Calprotectin (S100a8/S100a9): A Key Protein between Inflammation and Cancer." *Inflammation Research* 67, no. 10 (2018): 801-12.

5. Nakano, T., S. Goto, Y. Takaoka, H. P. Tseng, T. Fujimura, S. Kawamoto, K. Ono, and C. L. Chen. "A Novel Moonlight Function of Glyceraldehyde-3-Phosphate Dehydrogenase (Gapdh) for Immunomodulation." *Biofactors* 44, no. 6 (2018): 597-608.

6. Hamill, Kevin J, Sho Hiroyasu, Zachary T Colburn, Rosa V Ventrella, Susan B Hopkinson, Omar Skalli, and Jonathan CR Jones. "Alpha Actinin-1 Regulates Cell-Matrix Adhesion Organization in Keratinocytes: Consequences for Skin Cell Motility." *Journal of Investigative Dermatology* 135, no. 4 (2015): 1043-52.

7. Jin, Mei-Hua, Nan-Nan Yu, Ying-Hua Jin, Ying-Ying Mao, Lin Feng, Yue Liu, Ai-Guo Wang, Hu-Nan Sun, Taeho Kwon, and Ying-Hao Han. "Peroxiredoxin Ii with Dermal Mesenchymal Stem Cells Accelerates Wound Healing." *Aging (Albany NY)* 13, no. 10 (2021): 13926.

8. Takayama, Yoshiharu, and Reiji Aoki. "Roles of Lactoferrin on Skin Wound Healing." *Biochemistry and Cell Biology* 90, no. 3 (2011): 497-503.

9. Lu, Jacky, Miriam A Guevara, Jamisha D Francis, Sabrina K Spicer, Rebecca E Moore, Schuyler A Chambers, Kelly M Craft, Shannon D Manning, Steven D Townsend, and Jennifer A Gaddy. "Analysis of Susceptibility to the Antimicrobial and Anti-Biofilm Activity of Human Milk Lactoferrin in Clinical Strains of Streptococcus Agalactiae with Diverse Capsular and Sequence Types." *Frontiers in Cellular and Infection Microbiology* (2021): 905.

10. Gupta, Ashok Kumar, Devraj Parasar, Amin Sagar, Vikas Choudhary, Bhupinder Singh Chopra, Renu Garg, and Neeraj Khatri. "Analgesic and Anti-Inflammatory Properties of Gelsolin in Acetic Acid Induced Writhing, Tail Immersion and Carrageenan Induced Paw Edema in Mice." *PloS one* 10, no. 8 (2015): e0135558.

11. Witke, Walter, Arlene H Sharpe, John H Hartwig, Toshifumi Azuma, Thomas P Stossel, and David J Kwiatkowski. "Hemostatic, Inflammatory, and Fibroblast Responses Are Blunted in Mice Lacking Gelsolin." *Cell* 81, no. 1 (1995): 41-51.

12. Yang, Yu-wei, Chen-ning Zhang, Yi-jia Cao, Yu-xia Qu, Tian-yi Li, Tian-ge Yang, Di Geng, and Yi-kun Sun. "Bidirectional Regulation of I-Type Lysozyme on Cutaneous Wound Healing." *Biomedicine & Pharmacotherapy* 131 (2020): 110700.

13. Schröder, JM, and J Harder. "Antimicrobial Skin Peptides and Proteins." *Cellular and Molecular Life Sciences CMLS* 63, no. 4 (2006): 469-86.

14. Singer, Adam J, and Richard AF Clark. "Cutaneous Wound Healing." *New England journal of medicine* 341, no. 10 (1999): 738-46.

15. Kaijzel, EL, Pieter Koolwijk, MGM Van Erck, VWM Van Hinsbergh, and MPM De Maat. "Molecular Weight Fibrinogen Variants Determine Angiogenesis Rate in a Fibrin Matrix in Vitro and in Vivo." *Journal of Thrombosis and Haemostasis* 4, no. 9 (2006): 1975-81.

16. Duong, Leora, Steven P Gross, and Albert Siryaporn. "A Novel Antibacterial Strategy: Histone and Antimicrobial Peptide Synergy." *Microbial Cell* 7, no. 11 (2020): 309.

17. Brookes, John Deakin Lees, Joseph Swaminadan Jaya, Henley Tran, Ashish Vaska, Keagan Werner-Gibbings, Andre C D’Mello, Jennifer Wong, Chris N Lemoh, Alan C Saunder, and Ming Kon Yii. "Broad-Ranging Nutritional Deficiencies Predict Amputation in Diabetic Foot Ulcers." *The international journal of lower extremity wounds* 19, no. 1 (2020): 27-33.

18. Ding, Z., D. Gau, B. Deasy, A. Wells, and P. Roy. "Both Actin and Polyproline Interactions of Profilin-1 Are Required for Migration, Invasion and Capillary Morphogenesis of Vascular Endothelial Cells." *Exp Cell Res* 315, no. 17 (2009): 2963-73.

19. Mockenhaupt, Maja, Cecile Viboud, Ariane Dunant, Luigi Naldi, Sima Halevy, Jan Nico Bouwes Bavinck, Alexis Sidoroff, Jürgen Schneck, Jean-Claude Roujeau, and Antoine Flahault. "Stevens–Johnson Syndrome and Toxic Epidermal Necrolysis: Assessment of Medication Risks with Emphasis on Recently Marketed Drugs. The Euroscar-Study." *Journal of Investigative Dermatology* 128, no. 1 (2008): 35-44.

20. Suri, Bani Kaur, Navin Kumar Verma, and Artur Schmidtchen. "Toll-Like Receptor 3 Agonist, Polyinosinic-Polycytidylic Acid, Upregulates Carbonic Anhydrase Ii in Human Keratinocytes." *Acta Dermato-Venereologica* 98, no. 8 (2018).

21. Panayi, A. C., Y. Endo, M. Karvar, P. Sensharma, V. Haug, S. Fu, B. Mi, Y. An, and D. P. Orgill. "Low Mortality Oxidative Stress Murine Chronic Wound Model." *BMJ Open Diabetes Res Care* 8, no. 1 (2020).

22. Pechter, Patricia M, Joel Gil, Jose Valdes, Marjana Tomic‐Canic, Irena Pastar, Olivera Stojadinovic, Robert S Kirsner, and Stephen C Davis. "Keratin Dressings Speed Epithelialization of Deep Partial‐Thickness Wounds." *Wound repair and regeneration* 20, no. 2 (2012): 236-42.

23. Roth, Wera, Mechthild Hatzfeld, and Thomas M Magin. "Targeting the Palm: A Leap Forward toward Treatment of Keratin Disorders." *Journal of Investigative Dermatology* 132, no. 6 (2012): 1541-42.

24. Moll, Roland, Markus Divo, and Lutz Langbein. "The Human Keratins: Biology and Pathology." *Histochemistry and cell biology* 129, no. 6 (2008): 705-33.

25. Nielsen, Marcus MK, Eva Aryal, Elnaz Safari, Biljana Mojsoska, Håvard Jenssen, and Bala Krishna Prabhala. "Current State of Slc and Abc Transporters in the Skin and Their Relation to Sweat Metabolites and Skin Diseases." *Proteomes* 9, no. 2 (2021): 23.

26. D’Alessandro, Sarah, Andrea Magnavacca, Federica Perego, Marco Fumagalli, Enrico Sangiovanni, Mauro Prato, Mario Dell’Agli, and Nicoletta Basilico. "Effect of Hypoxia on Gene Expression in Cell Populations Involved in Wound Healing." *BioMed Research International* 2019 (2019).

27. Edwards, J Vincent, Phyllis Howley, Rachel Davis, Andrew Mashchak, and Steven C Goheen. "Protease Inhibition by Oleic Acid Transfer from Chronic Wound Dressings to Albumin." *International journal of pharmaceutics* 340, no. 1-2 (2007): 42-51.

28. Bangalore, N, J Travis, VC Onunka, J Pohl, and WM Shafer. "Identification of the Primary Antimicrobial Domains in Human Neutrophil Cathepsin G." *Journal of Biological Chemistry* 265, no. 23 (1990): 13584-88.

29. Cazander, Gwendolyn, Gerrolt N Jukema, and Peter H Nibbering. "Complement Activation and Inhibition in Wound Healing." *Clinical and Developmental Immunology* 2012 (2012).

30. Rafail, Stavros, Ioannis Kourtzelis, Periklis G Foukas, Maciej M Markiewski, Robert A DeAngelis, Mara Guariento, Daniel Ricklin, Elizabeth A Grice, and John D Lambris. "Complement Deficiency Promotes Cutaneous Wound Healing in Mice." *The Journal of Immunology* 194, no. 3 (2015): 1285-91.

31. Ganeshan, Kirthana, and Ajay Chawla. "Metabolic Regulation of Immune Responses." *Annual review of immunology* 32 (2014): 609.

32. Im, Michael JC, and John E Hoopes. "Energy Metabolism in Healing Skin Wounds." *Journal of Surgical Research* 10, no. 10 (1970): 459-64.

33. Donaldson, DONALD J, and JAMES T Mahan. "Fibrinogen and Fibronectin as Substrates for Epidermal Cell Migration During Wound Closure." *Journal of Cell Science* 62, no. 1 (1983): 117-27.

34. Martin, Paul. "Wound Healing--Aiming for Perfect Skin Regeneration." *Science* 276, no. 5309 (1997): 75-81.

35. Maione, Anna G, Avi Smith, Olga Kashpur, Vanessa Yanez, Elana Knight, David J Mooney, Aristidis Veves, Marjana Tomic‐Canic, and Jonathan A Garlick. "Altered Ecm Deposition by Diabetic Foot Ulcer‐Derived Fibroblasts Implicates Fibronectin in Chronic Wound Repair." *Wound repair and regeneration* 24, no. 4 (2016): 630-43.

36. Dimayuga, Paul, Jenny Zhu, Sumito Oguchi, Kuang-Yuh Chyu, Xiao-Ou Helen Xu, Juliana Yano, PK Shah, Jan Nilsson, and Bojan Cercek. "Reconstituted Hdl Containing Human Apolipoprotein a-1 Reduces Vcam-1 Expression and Neointima Formation Following Periadventitial Cuff-Induced Carotid Injury in Apoe Null Mice." *Biochemical and biophysical research communications* 264, no. 2 (1999): 465-68.

37. Vowinkel, Thorsten, Mikiji Mori, Christian F Krieglstein, Janice Russell, Fumito Saijo, Sulaiman Bharwani, Richard H Turnage, W Sean Davidson, Patrick Tso, and D Neil Granger. "Apolipoprotein a-Iv Inhibits Experimental Colitis." *The Journal of clinical investigation* 114, no. 2 (2004): 260-69.

38. Xu, Xiaohong Ruby, Yiming Wang, Reheman Adili, Lining Ju, Christopher M Spring, Joseph Wuxun Jin, Hong Yang, Miguel AD Neves, Pingguo Chen, and Yan Yang. "Apolipoprotein a-Iv Binds Αiibβ3 Integrin and Inhibits Thrombosis." *Nature communications* 9, no. 1 (2018): 1-18.

39. Karahan, Azize, Aysel AAbbasoğlu, Sevcan Avcı Işık, Banu Çevik, Çiğdem Saltan, Nalan Özhan Elbaş, and Ayşe Yalılı. "Factors Affecting Wound Healing in Individuals with Pressure Ulcers: A Retrospective Study." *Ostomy/wound management* 64, no. 2 (2018): 32-39.

| **Pair of Variables** | \| **Valid N** \| \| --- \| | \| **Spearman R** \| \| --- \| | \| **p-value** \| \| --- \| |
| --- | --- | --- | --- | --- | --- | --- |
| \| KRT9 & Q1 \| \| --- \| | 9 | -0.878669 | 0.001816 |
| \| KRT9 & Q2 \| \| --- \| | 9 | -0.794986 | 0.010445 |
| \| KRT9 & Q3 \| \| --- \| | 9 | -0.828459 | 0.005795 |
| \| KRT9 & Q4 \| \| --- \| | 9 | -0.652725 | 0.056677 |
| \| CAT & Q1 \| \| --- \| | 6 | -0.941124 | 0.005098 |
| \| CAT & Q2 \| \| --- \| | 6 | -0.941124 | 0.005098 |
| \| CAT & Q3 \| \| --- \| | 6 | -0.941124 | 0.005098 |
| \| CAT & Q4 \| \| --- \| | 6 | -0.880406 | 0.020599 |
| \| KRT1 & Q1 \| \| --- \| | 9 | -0.835510 | 0.005040 |
| \| KRT1 & Q2 \| \| --- \| | 9 | -0.683599 | 0.042330 |
| \| KRT1 & Q3 \| \| --- \| | 9 | -0.734236 | 0.024288 |
| \| KRT1 & Q4 \| \| --- \| | 9 | -0.607644 | 0.082612 |
| \| GAPDH & Q1 \| \| --- \| | 9 | -0.822152 | 0.006533 |
| \| GAPDH & Q2 \| \| --- \| | 9 | -0.584830 | 0.098112 |
| \| GAPDH & Q3 \| \| --- \| | 9 | -0.652636 | 0.056722 |
| \| GAPDH & Q4 \| \| --- \| | 9 | -0.372935 | 0.322899 |
| \| ACTN1 & Q1 \| \| --- \| | 9 | -0.798348 | 0.009892 |
| \| ACTN1 & Q2 \| \| --- \| | 9 | -0.773137 | 0.014549 |
| \| ACTN1 & Q3 \| \| --- \| | 9 | -0.848769 | 0.003807 |
| \| ACTN1 & Q4 \| \| --- \| | 9 | -0.621871 | 0.073765 |
| \| TPI1 & Q1 \| \| --- \| | 6 | -0.811679 | 0.049858 |
| \| TPI1 & Q2 \| \| --- \| | 6 | -0.753702 | 0.083523 |
| \| TPI1 & Q3 \| \| --- \| | 6 | -0.753702 | 0.083523 |
| \| TPI1 & Q4 \| \| --- \| | 6 | -0.927634 | 0.007666 |
| \| SLC4A1 & Q1 \| \| --- \| | 9 | -0.694567 | 0.037864 |
| \| SLC4A1 & Q2 \| \| --- \| | 9 | -0.644357 | 0.061033 |
| \| SLC4A1 & Q3 \| \| --- \| | 9 | -0.644357 | 0.061033 |
| \| SLC4A1 & Q4 \| \| --- \| | 9 | -0.736408 | 0.023655 |

**Table S4** Spearman correlation analysis of wound scrapings proteomic analysis and wound size measurements. Changes in protein expression in wound scrapings observed one week after treatment application (day 0 vs. day 7) were correlated with the throughout course of patients treatment score. Each protein expression level was paired against wound quotient from subsequent visits (Q1, Q2, Q3, Q4; day 7, day 14, day 21, day 49, respectively). Expression of 7 proteins showed negative correlation with the relative wound size, namely: KRT9 - keratin type I cytoskeletal 9; CAT - catalase; KRT1 - keratin type II cytoskeletal 1; GAPDH - glyceraldehyde-3-phosphate dehydrogenase; ACTN1 - alpha-actinin-1; TIP1 - triosephosphate isomerase; SLC4A1 - band 3 anion transport protein. P value cutoff point was <0.05, the rows that fulfill this cutoff point are marked in red.

**Table S5** Results of search for donor DNA in wound scrapings collected during subsequent visits on days 7, 14 and 21 by STR and ADS methods. Values represent percentage of donor’s DNA admixture, empty cell – donor’s DNA not detected, X - not studied due to lack of material, .

| ADSC-group | | | | | | |
| --- | --- | --- | --- | --- | --- | --- |
|  | Day 7 | | Day 14 | | Day 21 | |
| Sample | STR | ADS | STR | ADS | STR | ADS |
| 1ABC01013 |  |  |  |  |  |  |
| 1ABC01014 |  |  |  |  |  |  |
| 1ABC01016 |  |  |  |  | X |  |
| 1ABC01017 |  |  |  |  |  |  |
| 1ABC01018 |  |  |  |  | X |  |
| 1ABC01019 | 4.7 | 4.1 | 4.1 | 4.8 | X |  |
| 1ABC01023 |  |  |  |  |  |  |
| 1ABC01025 |  |  |  |  |  |  |
| 1ABC01026 | 3.8 | 3.1 |  |  |  |  |
| 1ABC01027 |  | 1.4 |  |  | 4.2 | 2.1 |
| 1ABC01028 |  |  |  |  |  |  |
| 1ABC01029 |  |  |  |  |  |  |
| 1ABC01030 |  |  | X |  | X |  |
| 1ABC01032 |  |  |  |  |  |  |
| 1ABC01033 | 4.3 | 3.1 |  |  |  |  |
| 1ABC01034 | 4.2 | 2.6 |  |  |  |  |
| 1ABC01035 | 2.2 | 1.3 |  |  |  |  |
| 1ABC01036 |  |  |  |  | X |  |
| 1ABC01037 |  |  |  |  |  |  |
| 1ABC01038 | 4.3 | 2.3 |  |  |  |  |
| 1ABC01039 |  |  |  |  |  |  |
| Fibrin gel group | | | | | | |
| 1ABC01004 |  |  |  |  |  |  |
| 1ABC01007 |  |  |  |  |  |  |

**Table S6** STR genotyping results of the donor DNA and DNA extracted from wound scrapings.

*Due to the size of the image the following table is additionally attached as a separate *.PDF file – “Table S6”

**Table S7** Comparison of mean area under peak (AUP) for donor specific “short” and “long” STR (short tandem repeats) alleles. Statistically significant difference was detected in the sample 1ABC01019 V2, p value <0.05 (bolded-out).

| **SAMPLE** | mean % AUP | | standard deviation | | t.test |
| --- | --- | --- | --- | --- | --- |
|  | short loci | long loci | short loci | long loci | var.diff |
| **1ABC01034 day 7** | 0,038551 | 0,048958 | 0,013426 | 0,031867 | 0,510626905 |
| **1ABC01033 day 7** | 0,043865 | 0,039271 | 0,019451 | 0,004346 | 0,578757782 |
| **1ABC01035 day 7** | 0,022057 | 0,022056 | 0,008784 | 0,00363 | 0,999768639 |
| **1ABC01019 day 7** | 0,05205 | 0,0394 | 0,015715 | 0,025624 | 0,235604899 |
| **1ABC01019 day 14** | 0,046073 | 0,024768 | 0,019333 | 0,00324 | **0,004118782** |
| **1ABC01026 day 7** | 0,040859 | 0,035085 | 0,022357 | 0,005977 | 0,75268254 |
| **1ABC01038 day 7** | 0,044768 | 0,038086 | 0,022783 | 0,011789 | 0,628333046 |
| **1ABC01027 day 21** | 0,040218 | 0,045292 | 0,015582 | 0,023726 | 0,802286027 |
| **All samples** | 0,042642 | 0,037768 | 0,018995 | 0,0219 | 0,28 |

**Table S8** Results of search for donor DNA in wound scrapings by ADS (amplicon deep sequencing) (sample, time point, coverage, A,G,%,C,%,T,%)

| ADSC group | | | | | | | | | | | | | | | | | | | | | | | | | | | |
| --- | --- | --- | --- | --- | --- | --- | --- | --- | --- | --- | --- | --- | --- | --- | --- | --- | --- | --- | --- | --- | --- | --- | --- | --- | --- | --- | --- |
|  | Day 7 | | | | | | | | | Day 14 | | | | | | | | | Day 21 | | | | | | | | |
| Sample | STR | coverage | REF [A] | ALT [G] | % | C | % | T | % | STR | coverage | REF [A] | ALT [G] | % | C | % | T | % | STR | coverage | REF [A] | ALT [G] | % | C | % | T | % |
| 1ABC01013 |  | 191814 | 190385 | 1007 | 0,5 | 321 | 0,17 | 101 | 0,05 |  | 3465 | 3445 | 16 | 0,5 | 0 | 0,00 | 4 | 0,12 |  | 210662 | 209620 | 578 | 0,3 | 319 | 0,15 | 145 | 0,07 |
| 1ABC01014 |  | 190356 | 189048 | 846 | 0,4 | 329 | 0,17 | 133 | 0,07 |  | 1654 | 1643 | 9 | 0,5 | 1 | 0,06 | 0 | 0,00 |  | 181173 | 180217 | 515 | 0,3 | 315 | 0,17 | 126 | 0,07 |
| 1ABC01016 |  | 198609 | 197607 | 558 | 0,3 | 321 | 0,16 | 123 | 0,06 |  | 200034 | 198864 | 568 | 0,3 | 502 | 0,25 | 100 | 0,05 | X |  |  |  |  |  |  |  |  |
| 1ABC01017 |  | 230312 | 229155 | 619 | 0,3 | 399 | 0,17 | 139 | 0,06 |  | 184063 | 182948 | 599 | 0,3 | 393 | 0,21 | 123 | 0,07 |  | 195033 | 194100 | 499 | 0,3 | 499 | 0,26 | 124 | 0,06 |
| 1ABC01018 |  | 192621 | 192621 | 1176 | 0,6 | 345 | 0,18 | 105 | 0,05 |  | 206311 | 205150 | 684 | 0,3 | 341 | 0,17 | 135 | 0,07 | X |  |  |  |  |  |  |  |  |
| 1ABC01019 | + | 9317 | 8936 | 378 | 4,1 | 0 | 0,00 | 3 | 0,03 | + | 9774 | 9304 | 465 | 4,8 | 3 | 0,03 | 2 | 0,02 | X |  |  |  |  |  |  |  |  |
| 1ABC01023 |  | 208069 | 206948 | 635 | 0,3 | 387 | 0,19 | 99 | 0,05 |  | 224151 | 222994 | 638 | 0,3 | 380 | 0,17 | 139 | 0,06 |  | 223643 | 222390 | 683 | 0,3 | 415 | 0,19 | 155 | 0,07 |
| 1ABC01025 |  | 188083 | 187038 | 612 | 0,3 | 328 | 0,17 | 105 | 0,06 |  | 161076 | 160232 | 467 | 0,3 | 296 | 0,18 | 80 | 0,05 |  | 234573 | 233308 | 752 | 0,3 | 383 | 0,16 | 130 | 0,06 |
| 1ABC01026 | + | 176199 | 170199 | 5538 | 3,1 | 299 | 0,17 | 104 | 0,06 |  | 216654 | 215424 | 719 | 0,3 | 358 | 0,17 | 153 | 0,07 |  | 227013 | 225443 | 1089 | 0,5 | 380 | 0,17 | 101 | 0,04 |
| 1ABC01027 |  | 3208 | 3154 | 46 | 1,4 | 6 | 0,19 | 2 | 0,06 |  | 195517 | 194284 | 738 | 0,4 | 394 | 0,20 | 101 | 0,05 | + | 6242 | 6106 | 129 | 2,1 | 1 | 0,02 | 5 | 0,08 |
| 1ABC01028 |  | 3466 | 3455 | 5 | 0,1 | 6 | 0,17 | 0 | 0,00 |  | 209476 | 208298 | 706 | 0,3 | 392 | 0,19 | 80 | 0,04 |  | 205904 | 204869 | 603 | 0,3 | 322 | 0,16 | 110 | 0,05 |
| 1ABC01029 |  | 3618 | 3605 | 11 | 0,3 | 0 | 0,00 | 2 | 0,06 |  | 163598 | 162726 | 473 | 0,3 | 316 | 0,19 | 83 | 0,05 |  | 156403 | 155591 | 495 | 0,3 | 227 | 0,15 | 90 | 0,06 |
| 1ABC01030 |  | 159692 | 158828 | 477 | 0,3 | 298 | 0,19 | 89 | 0,06 | X |  |  |  |  |  |  |  |  | X |  |  |  |  |  |  |  |  |
| 1ABC01032 |  | 239915 | 238030 | 1246 | 0,5 | 491 | 0,20 | 148 | 0,06 |  | 157511 | 156450 | 654 | 0,4 | 326 | 0,21 | 81 | 0,05 |  | 233582 | 232624 | 676 | 0,3 | 428 | 0,18 | 123 | 0,05 |
| 1ABC01033 | + | 10174 | 9855 | 317 | 3,1 | 1 | 0,01 | 1 | 0,01 |  | 185073 | 184017 | 662 | 0,4 | 303 | 0,16 | 91 | 0,05 |  | 235729 | 234203 | 986 | 0,4 | 415 | 0,18 | 124 | 0,05 |
| 1ABC01034 | + | 12914 | 12577 | 330 | 2,6 | 4 | 0,03 | 3 | 0,02 |  | 214915 | 213839 | 628 | 0,3 | 332 | 0,15 | 116 | 0,05 |  | 194111 | 193108 | 492 | 0,3 | 389 | 0,20 | 122 | 0,06 |
| 1ABC01035 | + | 15565 | 15350 | 206 | 1,3 | 4 | 0,03 | 5 | 0,03 |  | 163932 | 162287 | 453 | 0,3 | 345 | 0,21 | 152 | 0,09 |  | 245648 | 244375 | 696 | 0,3 | 439 | 0,18 | 138 | 0,06 |
| 1ABC01036 |  | 201683 | 200502 | 659 | 0,3 | 417 | 0,21 | 105 | 0,05 |  | 216019 | 214799 | 717 | 0,3 | 373 | 0,17 | 130 | 0,06 | X |  |  |  |  |  |  |  |  |
| 1ABC01037 |  | 209531 | 208480 | 534 | 0,3 | 400 | 0,19 | 117 | 0,06 |  | 188302 | 187026 | 586 | 0,3 | 579 | 0,31 | 111 | 0,06 |  | 224792 | 223557 | 632 | 0,3 | 462 | 0,21 | 141 | 0,06 |
| 1ABC01038 | + | 213070 | 207650 | 4883 | 2,3 | 397 | 0,19 | 140 | 0,07 |  | 215476 | 215206 | 701 | 0,3 | 458 | 0,21 | 111 | 0,05 |  | 207575 | 206399 | 641 | 0,3 | 419 | 0,20 | 116 | 0,06 |
| 1ABC01039 |  | 221846 | 220635 | 614 | 0,3 | 481 | 0,22 | 116 | 0,05 |  | 200334 | 199368 | 551 | 0,3 | 323 | 0,16 | 90 | 0,04 |  | 228734 | 227518 | 711 | 0,3 | 387 | 0,17 | 118 | 0,05 |
| Fibrin gel group | | | | | | | | | | | | | | | | | | | | | | | | | | | |
| 1ABC01004 |  | 14488 | 14429 | 38 | 0,3 | 16 | 0,11 | 5 | 0,03 |  | 20175 | 20109 | 47 | 0,2 | 10 | 0,05 | 9 | 0,04 |  | 18915 | 18811 | 69 | 0,4 | 24 | 0,13 | 11 | 0,06 |
| 1ABC01007 |  | 25634 | 25525 | 76 | 0,3 | 22 | 0,09 | 11 | 0,04 |  | 24450 | 24353 | 77 | 0,3 | 15 | 0,06 | 5 | 0,02 |  | 22249 | 22152 | 68 | 0,3 | 21 | 0,09 | 8 | 0,04 |

**Description S1: Detailed proteomic materials and methods**

For each scrapings sample part of about 50 μg was dissolved in 400 μl of 2% SDS, 100mM TEAB, MS-SAFE Protease, and Phosphatase Inhibitor, heated at 96 °C for 5 minutes, sonicated (Bioruptor setting: high) in 30 cycles 30sec ON/ 45 sec OFF, in a water bath with cooling (8°C), needle aspirated, and sonicated in the same way again. The sample was centrifuged, debris removed. Proteins were then precipitated with modified methanol/chloroform protocol.

Extracted proteins were reduced and alkylated with TCEP/MMTS. Samples were digested with protease mix LysC/Trypsin (Promega), for 4h, 37 °C, 6M urea, then diluted to urea concentration about 0.5M and digested overnight, as described previously. Peptide digest was purified with HLB 96 well plates (Waters), vacuum dried. 3 sets of samples were labeled with iTRAQ8-plex kits, allowing the comparison of 19 samples in 4 conditions. Each set was fractionated in High-pH protocol (HpH) using HLB 10 mg cartridge, pH=11, NH4CHO, MeCN gradient 2-80, collecting 6 fractions. Fractions were speed vac to dryness and then dissolved in 2% MeCN/0.1%TFA and analyzed by LC-MS/MS (liquid chromatography coupled to tandem mass spectrometry) using Nano-Acquity (Waters) UPLC system and QExative Orbitrap mass spectrometer (Thermo Fisher Scientific), by applying peptides to precolumn (nanoACQUITY UPLC Trapping Column Waters) using water containing 0.1% formic acid as a mobile phase and then transferred to nano‑column (nanoACQUITY UPLC BEH C18 Column, 75 μm inner diameter; 250 mm long; Waters) using an acetonitrile gradient (5–35 % AcN in 70 minutes) in the presence of 0.1% formic acid with the flow rate of 250 nl/min. Three washing runs ensuring lack of cross-contamination from previous samples preceded each analysis. Column outlet was directly coupled to the ion source of the spectrometer working in the regime of data-dependent MS to MS/MS switch. Peptides were eluted directly to the ion source of the mass spectrometer. Before each LC run, a blank run was performed to ensure no material was carried over from a previous analysis. Data was acquired in the m/z range of 300-2000. Data were searched, using MaxQuant 1.6.3.4. platform, against UniProt reference proteome database taxonomy: *Human,* 77027 entries*.* Variable modifications: oxidation (M), fixed modification: carbamidomethyl (C), peptide mass tolerance 20ppm, fragment ion tolerance 0.1Da, 2 missed cleavage, digestion: semispecific free N-term, PSM FDR 1, protein FDR 1 with a view to further statistical analysis with Scaffold Q+S 5.0.1. Identification results were analyzed using the Scaffold Q + S platform, compared with the Whitney-Mann test with Benjamin-Hochberg p-value 0.05 correction. On this basis, proteins that differentiated the time points and the conditions under study were selected. Change trends within a given group were also compared, extending the error range to p-value 0.05. Data was deposited in the PRIDE repository: access number PXD032099 (Reviewer account details: Username: reviewer_pxd032099@ebi.ac.uk; Password: r8FKHR9E).

**Description S2: SEARCH FOR DONOR DNA IN WOUND BIOPTATES - full description**

**Materials and methods**

**Short Tandem Repeats (STR) analysis**

We analyzed a total of 70 wound-swab samples (63 samples were collected from 21 recipients in the ADSC group at 3 time points (day 7, day 14, day 21), 6 negative controls from the fibrin gel group and 1 positive control (donor)). Genomic DNA was extracted using Maxwell RSC instrument (AS4500, Promega) and the Tissue DNA extraction kit (AS1610, Promega). The extracted DNA samples were split into two parallel procedures: STR (short tandem repeats) and ES/ADS (exome sequencing/amplicon deep sequencing).

For the STR analysis, DNA amplification was performed using an Applied Biosystems GeneAmp ® PCR System 9700 using the protocol described in the PowerPlex ® Fusion 6C System technical manual [1]. Briefly, amplification reactions contained 1.0 ng DNA. Separation of PCR products was performed using a 3500xl Genetic Analyzer (Applied Biosystems) with the internal lane standard WEN ILS 500 and allelic ladder provided with the kit. Final genotyping was conducted using GeneMapper-IDX v1.4 software (Thermo Fisher Scientific).

In the STR analysis, the presence of donor DNA was declared when min. two donor specific alleles were detected (using default settings of GeneMapper software) in the sample extracted from wound scraping. Donor alleles coinciding with stutter peaks (i.e. artefactual peaks known to occur at positions shorter by one repeat unit than a bona fide STR allele) present in the recipient were not considered.

The size of donor DNA admixture was estimated as follows: First, for each locus with donor admixture the area under the peak (AUP) of recipients alleles was averaged. In the case of homozygotes (amplification of two alleles of the same length), half of AUP was used. Then, for each donor allele detected in recipient we calculated the fraction of total DNA (Fd/t) it constituted. This was done by dividing AUP of the donor allele by the sum of mean recipient AUP and donor AUP. For the loci where donor was homozygous AUP was divided by 2.

**Next Generation Sequencing (NGS) analysis**

ES of the donor was conducted using Twist Core Exome kit (Twist Bioscience) with sequencing on NovaSeq 6000 (Illumina). Sequencing (paired-end reads, 2x100bp) yielded approx. 120mln reads, mean coverage was 122, ge10 (99.5%), ge20 (99.4%) and 99.88% target mapped. Bioinformatic analysis was carried out using in-home pipeline described previously [2] with the exception that Hg38 reference genome was used for alignment.

The ES analysis identified a homozygous variant in the *SMAD3* gene (rs35874463, Hg38 15:067165360-A>G, population frequency according to gnomAD= 0.03) which was selected as a marker of donor DNA. Subsequently, the ADS (*amplicon deep sequencing*) method was used to search for the presence of rs35874463 in the recipient samples. The designed primer sequences were: Forward: 5’-GGGACTTTGGTGCTGGTCTG-3’; Reverse: 5’-GGTCACGCTGCTCCTCTATG-3’; amplicon size was 430 bp, After PCR on DNA from wound scrapings samples libraries were prepared using Nextera XT DNA Library Preparation Kit (Illumina) according to the manufacturer’s instructions. The libraries were sequenced and analyzed in the same way as described for the ES of the donor sample. The size of admixture of donor DNA (Fd/r) was estimated by counting proportion of reads with G at rs35874463 among all reads from given sample. To determine the cut-off value for Fd/r in the ADS analysis we analyzed the results from sequencing DNA extracted from placebo wound scrapings. We found that for placebo samples the frequency of G at rs35874463 was always lower than 0.5% (the values for C and T were <0.3% and <0.12% for, respectively). Accordingly, in ADS analysis we used threshold of Fd/r =0.5% to declared that sample contained donor DNA.

**Assessment of DNA degradation**

In order to assess the degree of degradation of the detected donor DNA we analyzed whether the Fd/r showed bias towards higher values for shorter STR alleles (in STR analysis panels such as PowerPlex preferential amplification of shorter alleles is observed when DNA is significantly degraded). STR markers were divided into two groups according to their length. The group including the “short” STR loci consisted of: TH01, D8S1179, D16S539, D3S1358, vWA, D18S51, D1S1656, D19S433, D21S11, D2S441, D2S1338; and the group including the “long” loci: TH01l, D10S1248, D7S820, D13S317, FGA, CSF1PO, D5S818, Penta D, TPOX, Penta E, D22S1045, D12S391, SE33. The mean allele length for the first and the latter group was 171 bp (base pairs) and 370 bp, respectively. For each group of alleles the arithmetic mean and standard deviation were calculated for the Fd/r values and t-test with a two-tailed distribution and unequal variance was performed to statistically analyze the results. These analyzes were performed for individual recipients as well as for the pooled data.

**Results**

**Donor DNA in wound scrapings**

The results of both STR and ADS analyses are summarized in Supplementary Table 5**.** As can be seen from, out of 63 recipient samples from the ADSC group, admixture of the donor DNA was detected in 8 and 9 samples using STR and ADS method, respectively. Both methods detected the admixture in the same 8 recipient samples. Additionally, ADS showed donor admixture in one more sample (1ABC01027 from day 7) for which the STR results were negative. We noted, that in this sample donor’s DNA was also found on day 21 (but not on day 14) by ADS. Donor DNA was found in 7 out of 21 samples (33%) at the earliest time point day 7. At later time points (day 14 and day 21) only single samples were positive (a different one at each time point). The sample positive on day 21 (1ABC01027, positive by both STR and ADS) was also positive on day 7 (albeit only with ADS) but it was negative on day 14 (Supplementary Table 5). The estimated amount of the detected donor DNA was from 1.4 to 4.7 %. In two samples where donor DNA was detected later on day 14 or day 21 the amount present was apparently similar (1ABC01019) or higher (1ABC01027) than in the same patient on day 7 (Supplementary Table 5).

Analysis of the amount of donor DNA detected with STR loci with relatively long and short amplicon sizes did not show consistent differences (Supplementary Figure 3). In the analysis of individual samples only in one case (sample 1ABC01019 collected on day 14) we found significant difference (p= 0.0041) indicating a higher result with the short loci (0.046, SD 0.024) than the long loci (0.019, SD 0.0032). In other samples the trends were mixed – in 4 samples there was a trend for higher values for the short loci, in two – the opposite, in one - the values were virtually identical (Supplementary Figure 3). When all samples were pooled there was no significant difference between the mean values for the short and long loci (0.042 vs 0.038 respectively, p=0.28).

**Discussion**

By using two independent methods, we detected donor DNA admixture in 9 recipient samples. Majority of positive samples (n=7) were taken on day 7 and donor’s DNA presence at that time point was relatively high (7/21 or 33%). At later time points only two samples were positive indicating progression of clearance of donor’s material by that time. Given the sensitivity limit, in particular of the NGS method, we conclude that on day 14 and later majority of samples do not contain more than 0.5% of donor DNA. Nevertheless, it is interesting that in our limited series of patients, in one case donor’s DNA could clearly be detected as late as on day 21. Interestingly, in the estimated amount of donor’s DNA was actually higher on day 21 than day 7 and day 14. Whereas this can clearly be due to a chance, an intriguing possibility remains that this result reflects *in situ* proliferation of the applied cells.

The performed STR analysis allowed to tentatively evaluate the degree of degradation of detected donor DNA. The forensic STR panels like PowerPlex ® Fusion 6C used by us include a number of loci which differ in size of their amplicon sizes. In particular, in PowerPlex ® Fusion 6C the amplicon sizes range from 72–115bp for TH01 to 371–466bp for Penta E. When DNA is significantly degraded it is commonly observed that loci with relatively short amplicon sizes are preferentially amplified and yield more PCR product than loci with longer amplicon sizes which is reflected by differences in size (AUP) of the peaks on chromatogram. However, when we compared the estimates of the amount of donor DNA using the STR loci with short vs. long amplicon sizes we did not find significant differences apart from a single sample from day 14. In particular, there was no statistical difference in estimated fraction of donor DNA when pooled samples were analyzed after stratifying by STR amplicon size indicating that donor’s DNA, at least 7 days post treatment is not severely degraded. Given that the environment in the wound can be expected to promote degradation of biological molecules, our result is compatible, although clearly does not prove, with the presence of at least some living donor cells at the time of sampling.

It is reassuring that both methods used to detect donor DNA admixture in wound scrapings gave similar results with only a single discrepancy. Whereas the STR analysis with a dedicated forensic kit, such as PowerPlex ® Fusion 6C used by us, can be regarded as a standard approach to search for DNA admixture, the NGS approach is novel. This approach was based on the detection by NGS sequencing of a single rare DNA variant (which acted as a marker of donor DNA) in the amplicon generated by PCR on wound scraping DNA. Our results show that this approach had the sensitivity comparable (concordant result in 8 positive samples) or even superior (positive result in one sample which was negative in STR analysis) than the standard STR approach. The “marker” variant used by us was conveniently found by exome sequencing (ES) of the donor DNA. In addition to being rare it was selected to be homozygous in the donor, which should increase sensitivity of detection 2 times vs. heterozygous variants. Whereas the variant we used is specific to our donor, given general abundance of similar rare variants in the human exome [3], it is virtually certain that our approach can be readily applied in other studies.

**References:**

1. Promega Corporation Technical Manual #TMD045—PowerPlex ® Fusion 6C System Technical Manual. Promega Corporation. Madison, WI (01/15).

2. Ploski R, Pollak A, Müller S, Franaszczyk M, Michalak E, Kosinska J, Stawinski P, Spiewak M, Seggewiss H, Bilinska ZT. Does p.Q247X in TRIM63 cause human hypertrophic cardiomyopathy? Circ Res. 2014 Jan 17;114(2):e2-5. doi: 10.1161/CIRCRESAHA.114.302662. PMID: 24436435.

3. Karczewski, K.J., Francioli, L.C., Tiao, G. et al. The mutational constraint spectrum quantified from variation in 141,456 humans. Nature 581, 434–443 (2020). https://doi.org/10.1038/s41586-020-2308-7
