# Supplementary material for: Allogenic Adipose-Derived Stem Cells in Diabetic Foot Ulcer Treatment: Clinical Effectiveness, Safety, Survival in the Wound Site, and Proteomic Impact
Source: Int J Mol Sci. 2023 Jan 12;24(2):1472. doi: 10.3390/ijms24021472 (PMC9864558; doi:10.3390/ijms24021472)
Supplement: Supplementary file 1 [file ijms-24-01472-s001.zip › Supplementary Table S6 - after revision.pdf]

Table S6 – online quality

STR genotyping results of the donor DNA and DNA extracted from wound scrapings.

SAMPLE		AMEL	D3S1358	D3S1358	D1S1656	D1S1656	D2S441	D2S441	D10S1248	D10S1248	D13S317	D13S317	Penta E	Penta E	D16S539	D16S539
Donor		XX	17	18	14	15.3	13	14	14	15	8	11	11	14	12	13
Length			103	147	161	208	214	250	255	299	302	350	371	466	84	132
Mean length			125		184,5		232		277		326		418,5		108	
1ABC01034 day 7		XX	14	14	11	16.3	11	11.3	14	15	8	11	7	10	11	11
	peak area		425259		393190	351521	363169	319639	381451	372636	354490	361535	330886	348839	243960	
	admixture		17	18	14	15.3 (s)	13	14					11	14	12	13
	peak area		7404	4130	9449	47357	8645	9363					7421	39356	7300	7750
%			0,033649422	0,019053375	0,024748268		0,024696542	0,026692933					0,021368709	0,103781857	0,056466584	0,059739459
1ABC01033 day 7		XY	14	15	16	17	11.3	13	15	17	9	11	7	10	9	11
	peak area		105260	101872	104520	64663	82656	70979	94405	67276	66447	60544	80463	76369	121035	116920
	admixture		17												12	13
	peak area		1758												5195	2841
%			0,016691352												0,041836961	0,023321581
1ABC01035 day 7		XY	15	16	13	17.3	10	14	15	16	8	12	10	13	12	13
	peak area		318948	296390	333301	282799	299999	261410	216204	199213	270192	175337	149825	138660	348307	344536
	admixture			17		15.3										
	peak area			9177		6297										
%				0,028963597		0,020032003										
1ABC01019 day 7		XY	14	15	12	15	10	14	15	16	8	11	5	10	13	13
	peak area		402602	368484	342029	364036	374038	359860	297240	303226	348612	281880	212561	175634		267666
	admixture		17	18	14 (s)	15.3	13		14				11	14		12 (s)
	peak area		21504	15828	72813	14652	27612		34030				6008	4104		93428
%			0,05282928	0,039434837		0,039849382	0,069981574		0,101806063				0,030024162	0,020706201		
1ABC01019 day 14		XY	14	15	12	15	10	14	15	16	8	11	5	10	13	13
	peak area		263622	209050	218078	256253	202763	217907	175066	138243	205048	144112	108470	91776	355494	
	admixture		17	18	14 (s)	15.3									12 (s)	
	peak area		9197	4376	31792	5745									59420	
%			0,037457287	0,018179401		0,023650686										
1ABC01026 day 7		XY	16	18	14	16	11	14	15	16	11	11	7	14	12	13
	peak area		294297	265570	278358	244484	189263	246744	272664	302401	408384		221312	192268	317902	296315
	admixture								14 (s)		8		11			
	peak area								35880		9607		6313			
%											0,044934728		0,029624172			
1ABC01038 day 7		XY	15	16	16	17.3	10	10	13	16	9	12	19	20	12	13
	peak area		197780	146193	234943	229211	327679		173595	142904	165920	137933	160704	106312	260471	239412
	admixture		17	18			13				11 (s)		14			
	peak area		7660	6082			16537				14539		7698			
%			0,042639294	0,034155395			0,091680457						0,054516097			
1ABC01027 day 21		XY	14	18	12	17.3	10	14	13	15	8	11	7	16	11	11
	peak area		227546	156811	163540	152930	121076	186035	144083	145573	143537	132716	94918	72313	352214	
	admixture				14	15.3							11			
	peak area				5847	4966							2295			
%					0,035634622	0,030428735							0,026713848			

SAMPLE		D18S51	D18S51	D2S1338	D2S1338	CSF1PO	CSF1PO	Penta D	Penta D	TH01	TH01	vWA	vWA	D21S11	D21S11	D7S820	D7S820	D5S818	D5S818
Donor		18	22	18	24	11	11	13	13	6	8	16	16	31	31.2	10	10	11	12
Length		134	214	224	296	318	362	377	450	72	115	127	183	203	259	269	313	321	369
Mean length		174		260		340		413,5		93,5		155		231		291		345	
1ABC01034 day 7		15	16	17	17	9	11	9	9	6	7	16	18	32.2	33.2	10	10	9	13
	peak area	288569	346237	398468		344322	372776	501873		242859	231432	261995	216894	359253	298565	461125		375149	368124
	admixture	18	22		24			13						31	31.2 (s)			11	12 (s)
	peak area	16172	13686		10903			17780						12748	40056			6775	44331
%		0,048480851	0,041336317		0,051885199			0,066166387						0,037312275				0,01790379	
1ABC01033 day 7		13	16	17	23	11	14	12	13	7	7	15	15	29	29	7	10	10	11
	peak area	123743	92770	82775	80943	97654	103863	74859	108715	155932		109293		126490		75175	63008	98949	74961
	admixture			18	24							16		31					
	peak area			2689	3583							4787		3790					
%				0,031804419	0,04193488							0,080543801		0,05653763					
1ABC01035 day 7		15	15	16	19	8	12	9	13	6	9.3	15	17	27	30.2	10	10	11	12
	peak area	401053		214884	159713	225832	225609	145005	158453	219527	207273	233757	195064	231531	201111	352833		177096	168058
	admixture	18	22		24						8								
	peak area	7581	2580		4398						2433								
%		0,036428288	0,012702695		0,022942516						0,011272604								
1ABC01019 day 7		12	17	19	20	10	11	9	9	6	7	14	19	27	30	9	11	10	11
	peak area	349150	335262	241176	188536	302267	300029	411786		354224	305879	348042	320732	324835	313798	312667	257126	330538	257126
	admixture	18	22	18 (s)	24				13		8		16	31	31.2		10 (s)		12
	peak area	19266	8191	25585	10604				11574		21692		25109	7983	21210		42511		8463
%		0,053298734	0,023376342		0,047032733				0,053221868		0,061669938		0,069845005	0,024390505	0,062285901				0,027995832
1ABC01019 day 14		12	17	19	20	10	11	9	9	6	7	14	19	27	30	9	11	10	11
	peak area	244264	193861	128369	118412	152247	162916	232132		179006	154000	184892	181317	162527	172210	160595	138669	145175	129616
	admixture	18	22	18 (s)	24				13		8		16	31	31.2		10 (s)		12
	peak area	8725	10294	14632	5326				7734		7393		13380	5474	13704		18554		3947
%		0,038303243	0,044882094		0,041377757						0,042513916		0,068096975	0,031670451	0,075682392				0,027925076
1ABC01026 day 7		14	17	20	24	11	12	9	13	7	9	18	19	27	28	8	12	11	12
	peak area	298609	316035	319243	261176	42938	391069	202149	313763	169946	191169	188258	160504	249141	216363	185582	193959	256519	202661
	admixture	18								6 (s)	8 (s)				31.2				
	peak area	7468								8584	17323				6315				
%		0,023723752													0,026415189				
1ABC01038 day 7		12	17	20	25	10	11	9	10	6	9	17	19	28	30	8	11	11	12
	peak area	213176	174352	120932	131149	200838	191233	214279	198582	170500	150959	138211	131868	159221	188121	203036	157037	165307	173994
	admixture			18	24 (s)				13					31	31.2				
	peak area			6612	20013				5820					4389	4570				
%				0,049844519					0,02742043					0,024648995	0,025639443				
1ABC01027 day 21		15	15	24	25	10	12	9	12	9	9.3	15	19	30	32.2	10	12	12	13
	peak area	301766		150349	119648	173915	142519	130410	102486	134469	164020	126928	93388	131577	148755	108875	93623	152752	119909
	admixture		22	18			11 (s)				8 (s)			31	31.2 (s)				
	peak area		11508	4989			16799				8365			4115	13446				
%			0,070865996	0,035638896										0,028520734					

SAMPLE		TPOX	TPOX	D8S1179	D8S1179	D12S391	D12S391	D19S433	D19S433	SE33	SE33	D22S1045	D22S1045	DYS391	FGA	FGA	DYS576	DYS570
Donor		8	9	12	13	17	21	14	14	21	29.2	15	17	NA	19	20	NA	NA
Length		393	441	76	124	442	486	193	245			425	464		265	411		
Mean length		417		100		464		219		0		444,5			338			
1ABC01034 day 7		8	8	13	15	18	20	14	14.2	16	29.2	15	16					
	peak area	445161		312030	156936	362964	258183	306701	205690	280592	298680	264117	210450	brak	19	23		
	admixture			14 (s)		17 (s)	21			21					439523	386295		
	peak area			19551		36695	21362			5006								
%							0,064355876			0,020173526								
1ABC01033 day 7		8	8	14	15	17	18	13	13	21	25.2	11	11	10	23	26	16	17
	peak area	136882		61064	52268	106282	94684	145262		60807	55650	142892		83689	74005	111644	107031	112492
	admixture	9		12	13 (s)		21					17						
	peak area	2952		3505	6280		3453					3229						
%		0,041348592		0,058250652			0,033222368					0,04324071						
1ABC01035 day 7		8	11	13	13	17	22	14	15	15	30.2	12	15	10	22	24	18	19
	peak area	158357	131748	362215		318315	273517	197469	152797	162283	118805	143237	116063	330877	314926	298526	194475	191257
	admixture		9												19	20		
	peak area		2930												6001	8569		
%			0,019799638												0,019189261	0,027177722		
1ABC01019 day 7		8	8	15	15	20	22	12	15	25.2	30.2	15	16	10	21	22	19	18
	peak area	424105		419715		349474	363613	285550	271021	246613	198784	202252	204747	328204	345136	313792	253831	237414
	admixture		9	12	13	17	21 (s)		14 (s)	21	29.2 (s)				19	20 (s)		
	peak area		6594	13778	16050	10394	53195		54708	2207	30319				18272	51545		
%			0,030158269	0,061609181	0,071046778	0,02832635				0,00981301					0,052545609			
1ABC01019 day 14		8	8	15	15	20	22	12	15	25.2	30.2	15	16	10	21	22	19	18
	peak area	294558		281786		216362	224606	178423	147001	119610	104254	97968	98075	161954	182785	186898	81779	93361
	admixture		9	12	13	17	21 (s)		14 (s)						19	20 (s)		
	peak area		2905	12977	6905	5833	35323		23779						4940	15878		
%			0,019342939	0,084337428	0,046719171	0,025773583									0,026029934			
1ABC01026 day 7		8	8	10	14	19	20	13	15	20	27.2	15	15	10	23	25	18	18
	peak area	389567		160185	132972	279112	284856	186248	190543	142792	192008	346812		302928	215291	229597	318598	227438
	admixture			12	13 (s)				14 (s)						19	20		
	peak area			11447	26987				22931						7992	7140		
%				0,072437676											0,034682081	0,031099728		
1ABC01038 day 7		8	8	13	13	20	23	14.2	15	29.2	30.2	15	15	10	21	26	17	20
	peak area	300733			221452	139874	150999	120631	125722	107004	123364	229380		95905	224922	182188	135309	180204
	admixture		9							21					19	20 (s)		
	peak area		2902							3483					6799	18461		
%															0,032321705			
1ABC01027 day 21		8	10	13	14	22	22	14	15.2	13	35.2	15	15	10	22	24	19	19
	peak area	111782	121022	152090	132850	306344		122735	85739	137089	80943	209992		182028	170989	186305	160198	92886
	admixture														19	20		
	peak area														5598	15277		
%															0,030383457	0,078778284		
